# Mangrove Microbiomes as Drivers of Ecosystem Recovery and Restoration Success

**DOI:** 10.3390/microorganisms14061235

**Published:** 2026-05-30

**Authors:** Elijah Ige Ohimain, Robert Eugene Turner, Beth A. Middleton

**Affiliations:** 1 Department of Microbiology, Niger Delta University, Wilberforce Island, Amassoma 560103, Nigeria; 2 Department of Oceanography and Coastal Sciences, Louisiana State University, Baton Rouge, LA 70803, USA; euturne@lsu.edu; 3 Wetland and Aquatic Research Center, United States Geological Survey, 700 Cajundome Boulevard, Lafayette, LA 70506, USA; middletonb@usgs.gov

**Keywords:** coastal wetlands, ecosystem services, mangrove microbiome, mangrove restoration, microbiome

## Abstract

The microbes found in the rhizosphere, roots, leaves and stem surfaces and within the internal tissues of mangrove vegetation and their environment constitute the microbiome of the ecosystem. The organisms in the microbiome include bacteria, protozoa, fungi, algae, amoebas, and slime molds, which assist in maintaining and restoring mangrove ecosystems. This review explores the role of microbiomes in the maintenance of healthy mangrove ecosystems and in the successful restoration of degraded mangrove ecosystems. Microbes have important roles in several geomicrobiological cycles shaping mangrove ecosystems, including transforming nitrogen, phosphorus, carbon, sulfur and iron in biogeochemical cycles. Mangrove microbiomes contribute to the adaptation of vegetation to the harsh abiotic conditions in coastal areas, enhance nutrient uptake, produce plant-growth-promoting substances, and degrade the mangrove litter and the pollutants that can hinder restoration. Soil microbes function as biofertilizers, biopesticides, and bioremediation agents. The microbial diversity, composition, and functional capacity are important in the restoration of mangroves through their influence on voluntary recruitment following hydrologic restoration, on the establishment success of planted seeds and propagules, and on the survival of transplanted saplings and nursery-raised seedlings. The knowledge of the beneficial attributes of the microbiome can enhance the overall success of mangrove restoration. Identifying future needs, such as microbial inoculant validation, field-scale trials, and integration with hydrological restoration, are essential.

## 1. Introduction

Mangrove ecosystems consist of evergreen vegetation and other biotic and abiotic components occupying the coastlines of tropical and subtropical countries [1]. They are subject to daily tidal inundation. Microbes are microscopic lifeforms such as bacteria, archaea, fungi, viruses and protozoans which form the microbiome of this ecosystem. They are the unseen allies that assist in maintaining and restoring mangrove ecosystems. Intact mangrove ecosystems have a unique microbiome performing diverse functions that sustain the health and vitality of the ecosystem [2,3]. Changes in the composition of mangrove microbiomes are early warning signs of natural or anthropogenic impacts. Hence, recolonization by key microbial taxa, whether natural or assisted, has been considered for the restoration of impacted mangrove ecosystems. We review these attributes here. The objective of this narrative review is to synthesize the role of microbes in maintaining mangrove ecosystem function and supporting the restoration of degraded mangrove ecosystems, particularly through hydrologic recovery, propagule establishment, sapling transplantation, and nursery-based restoration.

### 1.1. Microbes and Mangroves—General Services

Microbes play important roles in virtually all the ecological services rendered by mangrove ecosystems, including income generation, food security, and cultural and psychological values [4]. Mangrove ecosystems also provide a habitat for wildlife, including fishery species, and protect the shorelines from coastal erosion and sea-level rise. In mangrove ecosystems, microbes mediate support services related to essential habitats for diverse species, soil formation, and nutrient cycling related to carbon, oxygen, sulfur, phosphorous, and nitrogen [5], supporting the resilience of the ecosystem [6,7,8]. The activities of anaerobic microorganisms, especially sulfur-reducing bacteria, play important roles in the genesis of mangrove soils and shape the redox conditions of the sediment. Sulfate-reducing bacteria mediate the trapping of heavy metals and other pollutants in sediments [9], while diverse species of microorganisms including heterotrophs are involved in mangrove litter degradation, the formation of organic matter, and nutrient cycling.

The services of mangrove ecosystems related to microbial contributions include their role in supporting nurseries for aquatic species, biodiversity, and stability of the ecosystem [9]. The growth and productivity of mangroves are enhanced by the nutrients made available via microbial cycling, and the biomass produced helps the system withstand sea surges. The partial degradation of organic matter under anaerobic conditions contributes to carbon burial and peat formation [7] and supports the trophic systems yielding seafood, biomass energy (fuel wood), building materials, fibers, medicinal herbs and fauna. These systems also contribute to the socioeconomic stability that has sustained rural coastal communities for centuries [2,4,10]. Microbial activities support the production of seafood such as shrimp, prawns, and crabs, which are vital for local livelihoods and nutrition and food security in coastal communities, because these microbes process the nutrients for primary and secondary producers in the food web [9].

### 1.2. Threats to Mangrove Ecosystems

Economic and other activities in mangrove areas have been a major threat to these ecosystems, including the conversion to crop agriculture, shrimp and fish farming, timber and fuel wood harvesting, sand mining and reclamation, urban encroachment, road construction, and sewage and garbage dumping [11,12,13]. Oil and gas exploration and production also impact mangroves; these activities include seismic exploration, canalization, the discharge of production effluents and oil spills [14,15]. Anthropogenic activities such as shrimp farming, dredging, oil spills, road construction, and hydrological disturbances alter microbial diversity, redox gradients, nutrient cycling, and plant–microbe interactions, which have profound effects on mangroves. For instance, activities such pipeline trenching, dredging, foundation words, the construction of fish and shrimp ponds, and road construction in mangrove ecosystems often lead to the disturbance of soils, exposing and causing the physicochemical and microbial oxidation of pyrites, resulting in acidification, which impacts the ecosystem greatly [5,14]. Mangrove ecosystems are increasingly under threat because they are located in or near urban areas [11] and industrial hubs (especially those focused on oil and gas exploration) [1,5,13,14,15] and where farming or aquaculture are thriving [1,12,13]. This review, therefore, focused on the roles of microbes in the maintenance of intact mangroves and the restoration of impacted mangroves. This review highlights microbial-assisted mangrove rehabilitation following hydrological restoration and is built on our growing knowledge of mangrove microbiomes [2,3], microbial ecosystem services [2,4] and mangrove restoration [1,2,12].

## 2. Mangrove Microbiome

The health, productivity, and resilience of mangrove vegetation is underpinned by the presence of diverse and unique microbial consortia, which perform a variety of functions [2,3]. Microbes found in the mangrove microbiome include bacteria, archaea, algae, protozoa, viruses, and fungi. Their presence is often overlooked because of their microscopic size, but their activities are essential for mangrove ecosystem survival and rehabilitation [2]. These microbes facilitate the adaptation of mangrove plants to estuarine conditions by enhancing water uptake, nutrient absorption, salt tolerance, and plant growth [16,17]. For instance, prolonged flooding and salinity exposure affect the physicochemical and microbial properties of mangrove soils [18,19]. Endophytic bacteria can increase salt tolerance and enhance the early establishment of mangrove seedlings. Microbes influence soil physicochemical properties such as anaerobiosis, low redox potential, and high sulfide, which are features that have profound effects on mangroves (discussed in more detail below).

### 2.1. Soil Biochemical Processes in Mangroves

Bacteria and fungi mediate chemical changes in soils with and without oxygen and have a strong effect on the availability of nutrients, trace metals, and heavy metals [3,16,20,21]. Oxidation occurs when a donor molecule gives up an electron and becomes oxidized. The acceptor molecule is reduced in the process. For example, sugars are oxidized to become carbon dioxide when electrons are given up and carbon, the acceptor, is reduced in the process. Organic carbon compounds are electron donors and are the source of most of the electrons either produced in the system or transported into the system by tides. Oxygen is a strong oxidant and makes the efficient degradation of plant materials possible [6]. The kinds of microbes and microbial metabolism change as oxygen becomes less available.

Carbon oxidation generates more chemical energy than the other oxidation–reduction (donor–acceptor) reactions discussed below. Oxidation, an aerobic reaction, generally takes place in the soil layer that is a few centimeters below the surface and where atmospheric oxygen penetrates into the soil by diffusion. Mangrove pneumatophores—the aerial roots protruding from the mud—aid in transferring oxygen to the soil. Diffusion processes limit oxygen availability. Sands are more porous and oxygen rich, but clays and silts are smaller in size and restrict the diffusion of oxygen. Oxygen is also transported downward from the leaves to the stems and to the roots and fibrous tissues, where microzones of oxygen can be a few millimeters thick around the roots. These plant microzones, called ‘rhizospheres’, are sites of intense chemical changes by bacteria and fungi [22] that are fueled by root exudates (carbon donors). Below or around the rhizospheres is an anaerobic zone with less (or no) oxygen.

The electron donor–acceptor reactions are dependent on how much ‘free energy’ is produced in a cascading series of reactions controlled by microbes. After oxygen depletion, the sequence of oxidation proceeds along the free-energy gradient, reducing NO_3_^−^, MnO_2_, Fe(OH)_3_, SO_4_^2−^, and CO_2_. The tendency to donate electrons is measured by the redox potential. The stronger the redox potential, then the more likely it is that an oxidation reaction will happen. Potential free energy is released only if both the electron donor and acceptor are present. The reactions cease when an electron donor–acceptor exchange yields no energy.

Nitrate (-1) accepts a carbon-based electron donor to reduce nitrate to N_2_ gas in what is known as denitrification. Microbes such as *Pseudomonas*, *Paracoccus*, and *Bacillus* carry out this process. Oxygen is the preferred electron donor for denitrification, but when oxygen is not present, nitrate is used; hydrologic moderations such as alternating wet and dry cycles or impoundment affect denitrification rates by influencing oxygen availability. Nitrification is a two-step process for converting ammonia or ammonium to nitrite and then nitrate. In the first step, ammonia or ammonium is oxidized and converted to nitrite. Bacteria frequently identified with this step are *Nitrosomonas*, *Nitrosococcus*, and *Nitrosospira*. The second step involves nitrite-oxidizing bacteria that oxidize nitrite to nitrate. The Nitrobacter genus is frequently identified as being involved in this second step, although other genera can also oxidize nitrite.

Under the anaerobic conditions created by microbes, the relatively insoluble manganese oxide (MnO_2_) ion is transformed to much more soluble manganous compounds (Mn^2+^), which can be toxic in acidic soils.

Ferric iron reduction to ferrous iron (Fe^+3^ to Fe^+2^) also uses carbon, which means that a scarcity of Fe^+2^ can limit organic matter decomposition. *Acidithiobacilsus ferrooxidans* bacteria are particularly important in these transformations. Fe^+3^ is highly insoluble and forms precipitates (Fe+^3^(PO_4_)_2_) that may bind with metals. A reduction to Fe^+2^ releases the iron phosphate in a dissolved phase. Adding iron may increase precipitation (because of iron scarcity) and trap phosphate and dissolved metals. Iron addition consumes the H_2_ and low-molecular-weight sugars that sulfate-reducing bacteria (SRB) or methanogenic bacteria use.

Sulfate (SO_4_) is reduced to hydrogen sulfide (H_2_S) under anaerobic conditions. Sulfate is the terminal electron acceptor and either organic matter or hydrogen gas acts as the electron donor. Sulfate is plentiful in seawater and depends on a more limited organic pool for the electron donor but yields little free energy. *Acidithiobacillus thioxidans* bacteria oxidize sulfur to sulfate, while SRB reduce sulfate to become hydrogen sulfide. The volatilization of H_2_S into the atmosphere gives off the ‘rotten egg’ smell characteristic of coastal mangroves. Its diffusion to areas with organic matter yields additional sulfate. It forms pyrite, diffusing downward, and stays within the anaerobic zone. When pyrite is exposed to oxygen, perhaps during wetland dredging or reclamation, it becomes H_2_SO_4_, creating acid sulfate soils, which plants cannot tolerate. Acid sulfate soils can be common in disturbed mangroves [5,14].

Methane production (methanogenesis) occurs after microbes reduce other electron acceptors. Hydrogen gas is the major electron donor, and formate or acetate may also donate hydrogen. The methane is either diffused to the atmosphere or converted to carbon dioxide gas when exposed to oxygen. Methane production may proceed more easily in freshwater systems if sulfate is absent.

**Figure 1:**
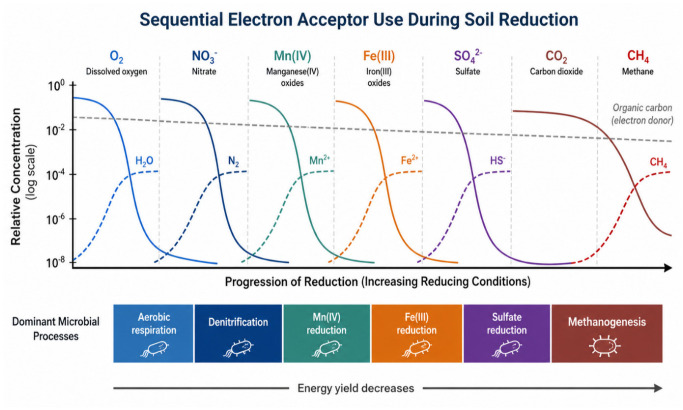
Schematic representation of the sequence of chemical transformations mediated by microbes that may occur in mangrove soils after flooding, beginning with oxygen consumption and moving through nitrate reduction, manganese reduction, iron reduction, sulfate reduction, and methanogenesis. Organic substrates (electron donors) gradually diminish as ammonium and phosphate increase (Redrawn and modified from Reddy and D’Angelo [23]). Reducing conditions increase with soil depth, resulting in the dominance of reduced forms of the chemical species present.

The suite of oxidation–reduction reactions (Figure 1) is mediated by the microbiomes amid hydrologic, geological, and other forces at a spatial scale, ranging from rhizospheres to trees, and temporal events such as a tidal cycle when water floods the marsh, or during infrequent storms or a decadal drought. Overharvesting the crabs that make the channels that irrigate mangrove soils, dredging channels, or constructing levees can add to these effects and cause the death of plants in one area or expansion into another.

### 2.2. Consequences

Mangrove plants, assisted by their microbiome, can resist the fast-changing conditions of daily and seasonal fluctuations in tides, fluctuating salinities, and tidal inundation [5]. These changes modulate the physicochemical environment of the soils in which mangroves are anchored, particularly salinity, oxygen, pH, redox potential, temperature, iron, and sulfate levels [24]. For instance, the microbial oxidation of minerals such as iron, pyrite, and other sulfides tends to scavenge oxygen and help to maintain the low-oxygen tension in mangroves. The activities of anaerobes such as sulfate-reducing bacteria in the breakdown of organic matter, including the reduction of sulfate to sulfides, results in the precipitation of heavy metals in unavailable forms and ameliorates acidity under anaerobic conditions. As a result of these dynamics, mangrove ecosystems are quite sensitive to even slight changes in topography and hydrology [5,14].

### 2.3. Common Microbes in Mangrove Soils

In a comprehensive review, Thatoi et al. [25] compiled some of the common groups and species of bacteria found in mangrove ecosystems, including N_2_-fixers, phosphate-solubilizers, sulfur/sulfate-reducers, methanogens and anoxygenic photosynthetic species (Table 1).

### 2.4. Metagenomic Discriminations

Recent metagenomic studies revealed several specific groups of microorganisms involved in diverse biogeochemical pathways (carbon, nitrogen and sulfur cycles) in mangrove ecosystems, including *Chloroflexi*, *Sulfurovum*, *Nitrospira*, *Anaerolinea*, and *Syntrophobacter* spp. [3,24,26]. Andreote et al. [16] used a 16S rDNA amplicon metagenomic method to detect the bacterial families Rhodobacteraceae, Planctomycetaceae, Burkholderiaceae and Desulfobacteraceae involved in the biogeochemical cycling of nitrogen, sulfur and methane in a mangrove ecosystem in Brazil. Wainwright et al. [27] reported guilds of microbes involved in nutrient cycling and/or the production of bioactive substances that promote the survival of their host mangrove plants. Das et al. [28] used metagenomic techniques to explore the taxonomic and the functional profile of the microbes involved in carbon cycling in mangrove soils in the Sundarbans and detected several genes regulating carbon metabolism, which are thought to play significant roles in the resilience and health of ecosystems. Ghose et al. [29] used a combined metagenomic approach based on shotgun and 16S amplicon sequencing to unravel the community structure and functional profiles of two mangrove ecosystems in Goa, India. They reported the dominance of bacterial groups from the phyla Chloroflexi, Proteobacteria, and Actinobacteria involved in the biogeochemical cycling of organic matter, carbohydrate metabolism, and the xenobiotic degradation pathways.

**Figure 2:**
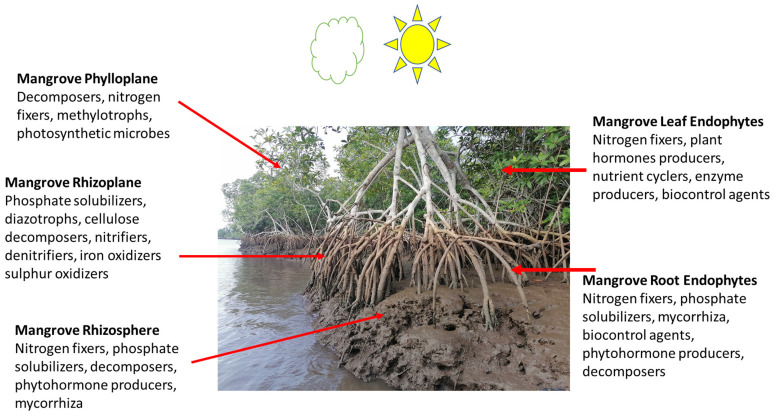
Functional groups of microbes associated with mangroves. Photo by Elijah Ohimain.

Various techniques can be used to determine the presence and role of the key microbes involved biogeochemical mineral cycling in mangroves. For instance, Costa et al. [24] used metagenomic approaches to predict the health of mangrove ecosystems. They observed that mangroves impacted by road construction had the lowest biodiversity, whereas mangroves recovering from road construction impacts had a mid-level biodiversity and intact mangroves had the highest level of soil microbial biodiversity. Intact mangroves are dominated by the genera *Desulfuromonas*, *Collinsella*, *Dorea*, *Dialister*, and *Desulfatiglans*. These genera are not present in impacted mangroves. The predominant phyla in all the mangrove types include Proteobacteria, Bacteroidetes, and Firmicutes. Generally, diverse functional groups of microbes associate with mangroves (Figure 2).

### 2.5. Epiphytic and Endophytic Microbes Associated with Mangrove Plants

Diverse microbes are found around the plant root (rhizosphere), and some occupy various parts of the plant surfaces such as the roots (rhizoplane) and leaves (phylloplane). Microbes found within the internal tissues of mangrove leaves, stems, and roots form the plant endosphere (Figure 2). Endophytic microbes form symbiotic relationships with mangroves, contributing to plant health and resilience by expressing metabolites that enhance mangrove growth and increase resistance to biotic and abiotic stress [30]. Shara et al. [31] demonstrated that three fungi (*Trichoderma harzianum*, *Lasiodiplodia theobromae*, and *Nigrospora sphaerica*) isolated from the phylloplane of *Rhizophora apiculata* were antagonistic to *Fusarium oxysporum*, a known plant pathogen.

## 3. Consequence of Disturbance to Mangrove Microbiomes

Mangrove disturbance affects their microbiomes. It causes shifts in microbial diversity, reduces beneficial taxa, increases acid-generating taxa, alters functional genes, and disrupts nutrient cycling. For instance, undisturbed mangrove soils are dominated by anaerobes, particularly the sulfate reducers, but when they become exposed as a result of dredging, aerobes such as the acid-producing *Acidithiobacillus* spp. become dominant [14,32]. If mangrove ecosystems are disturbed by development activities, particularly dredging, road construction, foundation work, and oil and gas exploration, some microbes (particularly *Acidithiobacillus* spp.) oxidize pyrites and other sulfur-containing minerals. This process can lead to the acidification that has further negative impacts on mangrove trees, fisheries, and other coastal life forms. The acidified soils can weather and void their sulfur content after about three decades, by which time the whole area will have lost its vegetation [14,32]. Attempts to restore such areas often fail due to the high soil acidity. High acidity levels often overwhelm any addition of lime intended to reduce the acidity. The weathered soils cannot support mangrove vegetation, due to the altered environmental quality, topography and hydrology, which typically is accompanied by the establishment of invasive species [14,32].

## 4. Microbial Activities Contributing to Mangrove Restoration

**Figure 3:**
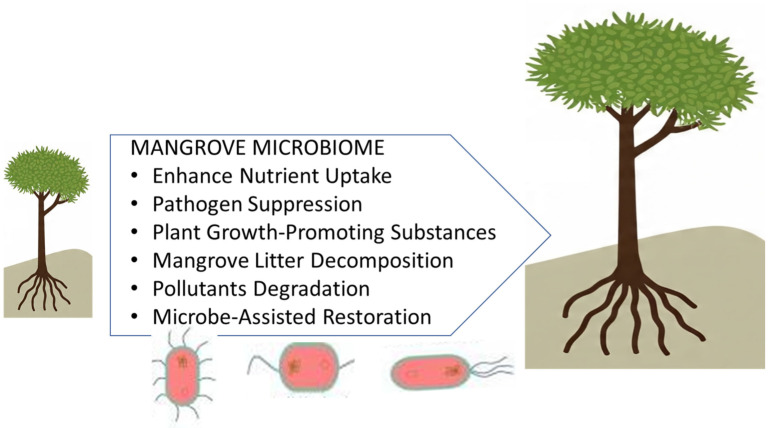
Microbiome role in assisting mangrove restoration.

The mangrove microbiome can enhance vegetation restoration (Figure 3). In the presence of a healthy microbiome, there are three common macro-propagation techniques used by humans to actively restore mangrove trees. These techniques include (1) volunteer recruitment resulting from hydrological restoration, (2) transplanting saplings and propagules, and (3) planting nursery-raised seedlings. However, natural recruitment following the restoration of the hydrological regime is quite effective and has even been considered as the first line of action [33,34]. The success of the mangrove restoration methods is linked to effective microbial modulation (a balanced microbiome) [2]. Recent studies have also considered the microbial mediated approach to mangrove restoration and rehabilitation [2]. These approaches seek to also restore the healthy microbiome, which could enhance the recovery of mangroves when combined with conventional mangrove restoration methods such as volunteer recruitment, the transplanting of propagules and saplings and the planting of nursery-raised seedlings [13,35]. All mangrove restoration techniques require an adequate supply of nutrients and the restoration of their microbiome and hydrology to be successful over time. Otherwise, the saplings grow initially but wither or even die before maturity [34]. Microbes play important roles in mangrove restoration by addressing some of the challenges affecting restoration, including hydrological changes, plant nutrition, pollutants such as oil spills or heavy metals, or the presence of debris or dead plants inhibiting the propagules from reaching the site. Microbes can play crucial roles in mangrove restoration by enhancing nutrient uptake, supporting plant growth, and facilitating the breaking down of organic matter and organic pollutants. The introduction of indigenous keystone microbial consortia, especially in disturbed mangroves, is considered an appropriate measure for their restoration and rehabilitation [36].

### 4.1. Production of Plant Growth Promoting Substances

Many beneficial microbes, particularly plant-growth-promoting microorganisms (PGPMs) including plant-growth-promoting bacteria (PGPB), cyanobacteria (PGPC), actinobacteria (PGPA) and fungi (PGPF), produce phytohormones such as auxins, gibberellins, and cytokinins, which directly influence plant root development, growth, and stress tolerance. Soil restoration through the inoculation of mangrove seedlings with these beneficial microbes can improve nutrient uptake, enhance mangrove growth and resilience, improve stress tolerance, and increase overall survival, making them more suitable for restoration projects. PGPMs enhance soil and plant health, mitigate abiotic stress, and improve ecosystem the services of mangroves [37].

Panda and Das [37] listed examples of PGPMs including PGPB, PGPC, PGPA and PGPF (Table 2). PGPMs also participate in mangrove leaf litter decomposition, promote nutrient cycling, enhance environmental remediation (organic and inorganic pollutants), promote pathogen inhibition, enhance soil stabilization, and form biofilms that increase stress resistance. Huang et al. [38] reported a case study where the diazotrophic bacteria *Novosphingobium* sp. N034 was inoculated into the seedlings of the mangrove plant *Kandelia obovata*. They found that the presence of these bacteria was related to increased plant height, ammonium level in the sediment, and nitrogen fixation rate. In India, Ravikumar et al. [39] demonstrated the enhancement of growth of *Ceriops decandra* and *Avicennia marina* seedlings when inoculated with three species of halophilic azotobacters, *Azotobacter beijerinckii*, *A. chroococcum*, and *A. vinelandii*. At a salinity of 30 ppt, *A. beijerinkii* inoculation significantly increased the root dry biomass by up to 76% in *Ceriops decandra*, and the shoot dry biomass of *A. marina* was, at a maximum, 56% higher than the uninoculated controls [39].

### 4.2. Mycorrhizal Fungi Enhancement of Mangrove Restoration

Mycorrhizal fungi may be important in mangrove restoration because they can encourage the establishment of plant species on dredged spoil disposal (placement) sites [40]. Vegetation establishment in dredged spoil can be limited by low nutrients, undesirable toxins (especially heavy metals), unsuitable moisture environments, and altered topography and hydrology [14]. Mycorrhizal fungi occur widely among plant species, including mangroves [40]. It is estimated that arbuscular mycorrhizal fungi (AMF) form beneficial relationships with more than 80% of vascular land plants [36,40]. Several species of mangroves form mutualistic associations with AMF in India, including *Aegiceras corniculatum*, *Avicennia marina*, *A. officinalis*, *Bruguiera cylindrica*, *Bruguiera gymnorrhiza*, *Ceriops tagal*, *Excoecaria agallocha*, *Kandelia candel*, *Lumnitzera racemosa*, *Rhizophora apiculata*, *Rhizophora mucronata*, and *Sonneratia alba* [36]. Kundu et al. [41] listed some fungal species that associate with mangrove species, including *Glomus*, *Claroideoglomus*, *Fusarium keratoplasticum*, *Fusarium solani*, *Penicillium chrysogenum*, *Rhizoctonia* spp., *Aspergillus fumigatus*, *Aspergillus niger*, *Gigaspora*, *Penicillium pinophilum*, *Acaulospora*, *Eutypella* sp., *Funneliformis*, *Cladosporium tenuissimum*, *Sclerocystis*, *Lasiodiplodia theobromae*, *Scutellospora*, *Rhizophagus*, and *Entrophospora*. Their association with plant roots helps to increase the absorptive area of the roots, which enhances water absorption and nutrient uptake including nitrogen and phosphorus, improves salinity and drought tolerance, stabilizes and aggregates soil, and protects roots from pathogens, resulting in increased survival rates, growth, and overall ecosystem resilience [36,41]. Mycorrhizal fungi can enhance mangrove growth rates by 2- to 80-fold in hardwood seedlings [40]. Due to their role in improving growth, mycorrhizal associations with mangroves have net positive effects on carbon sequestration, ecosystem productivity, and sediment stabilization [41]. Restoration is more successful using tailor-made mycorrhizal inocula specific to the local mangrove species and environmental conditions [41].

### 4.3. Bioremediation to Prepare the Site for Mangrove Restoration

Removing soil pollutants such as crude oil, soil acidifiers, and heavy metals can improve the outcomes of mangrove restoration. The mangrove microbiome is well suited to tackle pollutants via bioremediation in preparation for restoration. Major oil and gas resources exist underground in mangrove ecosystems, including the Gulf of Guinea, the Gulf of America (Gulf of Mexico), the Orinoco Delta, and the Malay Peninsula. Oil spills are major challenges for mangroves and their restoration [12,13,15]. For instance, oil pollution above 80 ppm in the soil has resulted in the death of planted seedlings [34]. Oil spill clean-up can therefore support mangrove restoration in impacted sites. Oil spill remediation in mangrove areas is quite challenging due to fluctuating tides and mangrove root systems that hinder clean-up. Oil can also become trapped in backswamp (swamp behind levees), penetrate the soil strata, or sink into the sediments when the volatile fractions have been lost [5]. The techniques used for oil clean-up include mechanical, chemical, and microbiological approaches. All of these methods have strengths and weaknesses and are commonly used in combination with each other. For instance, it is common to use physical means such as booming, trenching, and scooping to recover the bulk of the oil spills. Chemicals such as dispersants or microbiological methods are also used to clean the residual oil to meet regulatory requirements before attempting mangrove restoration. Mechanical methods such as cool water jetting and low-pressure washing have been used to clean oil spills in mangroves. For instance, in Ogoniland in the Niger Delta, Nigeria, in the Gulf of Guinea, oil spill bioremediation was conducted with bacteria before tree planting [42,43]. Soil bacteria from the families Acidithiobacillaceae, Alcanivoracaceae, Desulfobacteraceae and Oceanospirillaceae [42] and the phyla Firmicutes and Proteobacteria [43] are involved in the microbial degradation of pollutants and help to prepare the soil for successful mangrove restoration. These microbes carry out the biodegradation of pollutants, oil spills, garbage, and sewage [22]. Diesel spills may be degraded by bacterial inoculates [44]. The microbial breakdown of pollutants in mangrove ecosystems can enhance ecosystem health and support their restoration [45,46].

Acidity and heavy metal pollution, especially when mangrove ecosystems are disturbed, present significant challenges for mangrove restoration. Construction activities, including dredging, road construction, and seismic line and pipeline installation, disturb mangrove soils by exposing their pyritic layers. These activities typically result in the microbial and physicochemical oxidation of pyrites, thereby releasing copious acids, which are related to the release of heavy metals [14,35]. The activities of sulfate-reducing bacteria in mangrove ecosystems may reduce acidity because they change sulfate to hydrogen sulfide, which immobilizes heavy metals in non-available forms in mangrove sediments [47], but the hydrogen sulfide produced can be toxic to plants. Heavy-metal tolerant bacteria are present in mangrove ecosystems and are thought to play significant roles in the detoxification and amelioration of heavy-metal stress [48,49].

### 4.4. Microbial-Assisted Mangrove Restoration

There are several practical applications illustrating how microbiomes are important to mangrove restoration. Mangrove microbiomes are important in the natural restoration of mangroves disturbed by shrimp farming in Brazil [50]. The inoculation of arbuscular mycorrhizal fungal on *Kandelia obovata* seedlings boosts the supply of phosphorus and other nutrients [51]. A high percentage (97%) of arbuscular mycorrhizal fungi colonization occurred in self-recruited *Avicennia lanata* following natural hydrologic restoration, indicating that this microbiome was related to recovery in Mahakam Delta, East Kalimantan, Indonesia [52]. Seedlings containing nitrogen-fixing symbioses and mycorrhiza have been explored in mangrove restoration [45]. Uma et al. [46] discussed the cultivation and mass production of mangrove microbes for use as a plant-growth promoter with practical applications as microbial inoculants, biofertilizers, and biopesticides to enhance the productivity and improve the health of the ecosystem without the need for agrochemicals. Such beneficial microbes are either applied to the soil as a biofertilizer or to nursery-raised seedlings before transplanting [53].

## 5. Conclusions

The microbes forming the mangrove microbiome improve the restoration potential of damaged coastal ecosystems and ultimately support ecosystem and cultural services related to regulating, provisioning, and supporting mangrove ecosystems. Their involvement in geomicrobiological processes affects important cycles such as sulfur, iron, phosphorus, and nitrogen cycling. The microbes influence or enhance the adaptation of mangrove plants to abiotic environmental conditions affected by extremes in hydrology, salinity, acidity, and low oxygen levels. The bacteria facilitate the immobilization of heavy metals to bio-unavailable forms in the mangrove sediments. The mangrove microbes enhance mangrove restoration because their presence can enhance nutrient uptake and phytohormones, degrade mangrove litter and organic pollutants, immobilize heavy metals, and ameliorate high acidity levels. The applications of the microbes in biofertilizers, biopesticides, and bioremediation agents can facilitate the macro-propagation of mangrove vegetation for restoration projects. The logical next steps for restoration technology include inocula development, field trials and optimization.

## Figures and Tables

**Table 1:** Major functional groups of microbes reported from mangrove soils, adapted from Thatoi et al. [25].

Groups of Microbes	Examples
N_2_-fixers	*Azotobacter*, *Azospirillum*, *Rhizobium*, *Klebsiella*, *Clostridium*
Phosphate-solubilizers	*Pseudomonas*, *Bacillus*, *Xanthobacter*, *Paenibacillus*, *Kluyvera*, *Vibrio proteolyticus*, *Enterobacter*, *Chryseomonas*
Sulfur /sulfate-reducers	*Desulfotomaculum*, *Desulfovibrio*, *Desulfococcus*, *Desulfosarcina*
Methanogens	*Methanoccoides methylutens*
Anoxygenic photosynthetic species	*Chromatium*, *Chloronema*, *Thiopedia*, *Beggiatoa*, *Leucothiobacteria*

**Table 2:** Plant-growth-promoting microbes.

Group	Species
PGPB	*Azotobacter*, *Azospirillum*, *Bacillus*, *Alcaligenes*, *Acinetobacter*, *Arthrobacter*, *Rhizobium*, *Clostridium*, *Burkholderia*, *Enterobacter*, *Pseudomonas*, *Flavobacterium*, and *Paenibacillus*
PGPC	*Anabaena*, *Nostoc*, *Microcoleus*, *Calothrix*, *Oscillatoria*, *Lyngbya*, and *Aphanothece*
PGPA	*Actinophytocola*, *Pseudonocardia*, *Nocardiopsis*, and *Streptomyces*
PGPF	*Fusarium*, *Aspergillus*, *Gliocladium*, *Penicillium*, *Humicola*, *Trichoderma* and *Phoma*

Plant-growth-promoting bacteria (PGPB); plant-growth-promoting cyanobacteria (PGPC); plant-growth-promoting actinobacteria (PGPA); plant-growth-promoting fungi (PGPF).

## Data Availability

No new data were created or analyzed in this study. Data sharing is not applicable to this article.
